# Stress and psychological trauma as predictors of cancer-related fatigue in breast cancer patients (SaFE study)—study protocol of a prospective follow-up study

**DOI:** 10.3389/fpsyg.2025.1691485

**Published:** 2025-10-09

**Authors:** Hanna Hofmann, Thorsten Koch, Cosima Brucker, Peter Radermacher, Markus Müller, Christiane Waller, Barbara Stein

**Affiliations:** ^1^Department of Psychosomatic Medicine and Psychotherapy, General Hospital Nuremberg, Paracelsus Medical University, Nuremberg, Germany; ^2^Department of Gynecology and Obstetrics, General Hospital Nuremberg, Paracelsus Medical University, Nuremberg, Germany; ^3^Anesthesiological Pathophysiology and Process Engineering, University Hospital, Ulm, Germany; ^4^Department of Intensive Care and Hyperbaric Medicine, University Hospital, Angers, France

**Keywords:** cancer-related fatigue, breast cancer, acute stress, chronic stress, global stress index

## Abstract

**Introduction:**

Cancer-related fatigue (CRF) is a common symptom of cancer and/or its treatment. Most cancer patients are affected during treatment, as well as years thereafter. Around a third of survivors report suffering from CRF. Those affected are often restricted in their everyday life. Acute and chronic stress are factors that increase a person’s vulnerability to develop CRF. In previous studies different instruments measuring acute and chronic stress related to CRF were used. However, a global instrument to determine individual stress load is lacking.

**Methods:**

Therefore, a developed global stress index (GSI) combining specific measuring instruments for acute and chronic stress is validated on an oncological sample and its influence on fatigue is examined. It is hypothesized that individuals with a high global stress load measured by the GSI report higher levels of CRF. The data will be collected using questionnaires in participants suffering from breast cancer with a curative treatment approach. Participants will be surveyed during tumor-specific therapy and six months later. They receive a consultation if fatigue symptoms are strongly pronounced. The study is registered at Deutsches Register Klinischer Studien, no. DRKS DRKS00027864.

**Discussion:**

This will be the first study using the GSI as a valid measure for surveying longitudinally acute and chronic stress load in an oncological sample in relation to CRF symptom development. The GSI may help to identify tumor patients with high levels of stress in good time and thus prevent chronic fatigue.

## 1 Introduction

Fatigue is defined as a distressing and persistent feeling of physical, emotional or cognitive tiredness or exhaustion that is not related to recent activity and interferes with a person’s normal level of functioning (Fukuda, 1994; National Comprehensive Cancer Network, 2018). Symptoms of fatigue can be varied and range from feelings of lack of energy or exhaustion to depressive symptoms such as loss of drive or interest and difficulty concentrating. Those affected describe restrictions in their family and social life. These include difficulties in coping with everyday life, difficulties at work and a higher probability of unemployment, depressive and anxiety symptoms, lower quality of life and higher mortality rates (Sharpe et al., 1991; Curt et al., 2000; Scott et al., 2011; Weis, 2011; Horneber et al., 2012; Thong et al., 2020).

Fatigue is one of the most common side effects of cancer and cancer therapies (cancer-related fatigue—CRF). It occurs across various tumor entities as well as during or after therapy (Weis, 2011; Bower, 2019). During therapy, 50–90% of cancer patients are affected by CRF (Minton et al., 2013; Thong et al., 2020). In a recent meta-analysis, the average prevalence was 52% (Ma et al., 2020). In the long term—up to ten years after completion of therapy—around 30% of patients suffer from CRF (Kuhnt et al., 2011; Wu et al., 2019; Fabi et al., 2020). The high symptom burden, the effects on participation in everyday life described above and the reduced quality of life of those affected show the need for timely recognition of risk factors of developing CRF and specific treatment. The effectiveness of non-pharmacological forms of treatment, such as psychosocial (especially psychoeducation) and mindfulness-based (especially yoga) interventions or physical training, has already been proven and recommended (Bower, 2014; Fabi et al., 2020; Haussmann et al., 2022).

The risk factors that increase a person’s vulnerability to develop CRF include, in particular, psychosocial factors such as a history of depression, an anxious personality structure, loneliness, acute stress and traumatic childhood experiences (Lockefeer and De Vries, 2013; Bower et al., 2018; Bower, 2019). Women with breast cancer who perceive their tumor disease as a threat were also found to be associated with CRF (Levkovich et al., 2015). Numerous studies indicate that childhood experiences of abuse, maltreatment and neglect as well as cumulative stress over the lifespan are associated with CRF (Fagundes et al., 2012; Witek Janusek et al., 2013; Han et al., 2016; Bower, 2019). Women with breast cancer who had experienced trauma in childhood suffered significantly more frequently and more intensely from CRF than non-traumatized women with breast cancer. Similar associations were found for cumulative stress experience and CRF: women with breast cancer affected by CRF showed more acute and chronic stressors than women with breast cancer without CRF (Bower et al., 2014).

These previous studies have used different measurement instruments, e.g., traumatic childhood experiences were recorded with the Childhood Trauma Questionnaire (CTQ; Bernstein et al., 1994) and stress experienced during cancer with individual scales like the Perceived Stress Scale (PSS-10; Cohen et al., 1983). Since traumatic life events are part of the cumulative stress experience over the life span and symptoms such as depression and anxiety are also an expression of the cumulative stress load, it would be desirable to be able to define a global stress load measure across all the dimensions mentioned individually for a patient. In preliminary work by our working group (Maier et al., 2021), such a global stress index (GSI) using validated measurement scales in a sample of 192 healthy soldiers as part of a study funded by the Federal Ministry of Defense [Bundeswehreinsatz und Stress-Studie (BEST)] has been developed. For this purpose, validated measurement instruments of acute and chronic stress from childhood to adulthood were tested and converted into a GSI using structural equation modeling. Validation is currently underway in a healthy sample with an elevated cardiovascular risk profile.

### 1.1 Aims of the prospective study

It is important to identify risk factors at an early stage and offer early treatment to the patients with high psychosocial stress load. Therefore, the present study examines whether the GSI can be validated in an oncological sample in order to develop a global stress measure for acute and chronic stress for tumor patients as well. It is assumed that the GSI is also suitable as a stress measure for oncology patients. Furthermore, it is investigated which effects cumulative stress experience has on CRF in women with breast cancer with a curative treatment approach. It is assumed that women with a higher GSI score report a higher level of CRF.

## 2 Method and analysis

The study is being implemented as a monocentric, longitudinal study at the General Hospital Nuremberg, Paracelsus Medical University. To develop a GSI for oncological patients, women with breast cancer are asked about acute and chronic stressors and fatigue. For ethical reasons, a psychotherapeutic counselling session was offered in the presence of fatigue.

### 2.1 Recruitment

In close cooperation with the Breast Center at the General Hospital Nuremberg *N* = 200 women will be recruited. All patients discussed in the tumor board conference who meet the inclusion criteria will be identified by the study staff and invited to participate in the study during their subsequent medical consultation. After verifying inclusion and exclusion criteria, patients are informed about the study’s purpose, potential benefits, and risks before signing the consent form. After agreeing to take part in the study (written consent), the women receive a set of questionnaires during tumor-specific therapy, about 8 weeks after being included into the study. If a clinically relevant problem (e.g., severe psychiatric illness, self-endangerment or endangerment of others) is identified during the recruitment, the patient will be offered a psychological treatment (inpatient or outpatient treatment in the Department of Psychosomatic Medicine and Psychotherapy or in the Department of Psychiatry and Psychotherapy).

### 2.2 Inclusion and exclusion criteria

Inclusion criteria are a curative treatment approach (including chemotherapy and/or radiation and/or hormone therapy and/or immunotherapy) in women diagnosed with breast cancer. Exclusion criteria are palliative disease/treatment situation, severe psychiatric illness, untreated physical illness, malnutrition, cognitive impairment and insufficient knowledge of the German language.

### 2.3 Patient survey

The GSI survey consists of the following questionnaires that will be carried out on the participants:

- Perceived Stress Scale (PSS-4) (Cohen et al., 1983)
- Trier Inventory for Chronic Stress (TICS) (Schulz et al., 2004)
- Hospital Anxiety and Depression Scale (HADS) (Herrmann-Lingen et al., 1995)
- Childhood Trauma Questionnaire (CTQ) (Bader et al., 2009)
- Posttraumatic Diagnostic Scale Checklist (PDS) (Ehlers et al., 1996)
- The Deployment Risk and Resilience Inventory (DRRI-2) (Vogt et al., 2013)

The EORTC QLQ-FA12 (The European Organisation for Research and Treatment of Cancer Quality of Life Group, 2016) is used to record CRF. In addition, socio-demographic variables such as gender, age, education and marital status are surveyed. Completing the questionnaires will take about 45 min in total. Patients with high fatigue symptoms will receive a counselling session and, if necessary, further treatment.

### 2.4 Data collection

**Figure 1:**
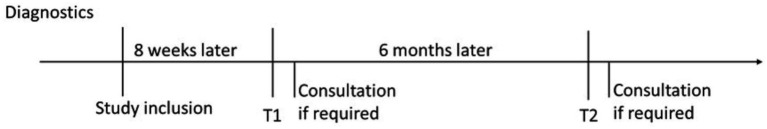
Study procedure and consultations.

The women receive the questionnaire by post. They answer it during the tumor-specific therapy (T1) and six months later (T2) (Figure 1). The women return the questionnaire in a prepaid envelope. If we detect fatigue after the evaluation, we contact the women for a consultation for ethical reasons.

### 2.5 Consultation

If fatigue is less pronounced, the women receive written information on fatigue and its treatment options. If patients with high fatigue symptoms are identified, they receive a counselling session by the psychosomatic consultation liaison services and, if necessary, further treatment. The counselling session takes place by telephone or in the clinic. After a detailed medical history, which includes questions about the status of tumor-specific therapy, physical and mental illnesses, the Cella et al. (1998, 2001) criteria are used to confirm the presence of CRF. The recommendations to patients are based on the guidelines that refer to CRF (Fabi et al., 2020; Howell et al., 2015). In addition to physical exercise, mindfulness-based and psychotherapeutic treatment approaches are recommended and patients are referred for further inpatient or outpatient treatment.

### 2.6 Data analysis

The sample size of *N* = 200 will be calculated over the A-priori Sample Size Calculator for Structural Equation Models^1^ analogous to the validation study of the GSI by Maier et al. (2021).

Data are evaluated in anonymized form. The women are given a pseudonym to complete the questionnaires. As the DRRI-2 with the reformulated item has not yet been examined for psychometric properties, construct validity will be evaluated using confirmatory factor analysis, and internal consistency will be assessed through reliability analyses (Cronbach’s *α*). Subsequently, the GSI is tested using the open-source software R (R Core Team, 2017). Item distribution and the variance between the items are considered first. The multivariate normal distribution in the whole item set is examined. In Maier’s study (Maier et al., 2021), the stress index was tested on a healthy sample using an iterative procedure at item level. Various models were tested. Finally, a hierarchical multilevel model with a g-factor was selected. The g-factor was the highest order to which the latent variables (perceived stress, HADS and CTQ) load directly. At a third level are the subscales of the CTQ, which have an additional informative influence on the GSI. The manifest variables (DRRI-2 and PDS) are at the lowest hierarchical level. In the present study, this proposed method is now to be reviewed and tested to see whether the factor structure is also evident in the oncological sample. It is also investigated whether the combination of all scales provides an additional value.

Correlations between the GSI and CRF are calculated using Pearson correlations. Predictions of GSI on fatigue are determined using regression models. The course of the fatigue symptoms is calculated with pre-post comparisons either using parametric or non-parametric tests depending on the distribution of the collected data.

## 3 Discussion

To the best of our knowledge, this will be the first study to combine instruments of acute and chronic stress and transfer them into a Global Stress Index (GSI) in an oncological sample. Study patients receive the questionnaire set during oncological therapy and 6 months later, measuring acute and chronic stress as well as CRF. Women suffering from severe fatigue symptoms will receive a psychological counselling session and, if necessary, further treatment.

In the following studies investigating stress and CRF, several individual measurement instruments were used: Han and colleagues (Han et al., 2016) examined breast cancer patients in a prospective longitudinal study during and 6 weeks after radiotherapy and showed that women who had experienced childhood trauma (CTQ; Bernstein et al., 1994) suffered significantly more from CRF than women who had not experienced childhood trauma before. The traumatized women also reported higher levels of depression (Inventory of Depressive Symptomatology-Self Reported (IDS-SR); Rush et al., 1996) and acute stress (PSS-10; Cohen et al., 1983). Witek Janusek et al. (2013) showed that childhood trauma (CTQ; Bernstein et al., 1994) increased vulnerability to fatigue, perceived stress (PSS-10; Cohen et al., 1983) and depressive symptoms (CES-D; Radloff, 1997), lower quality of life and immunological dysregulation in breast cancer patients. Women who were neglected or abused (CTQ; Bernstein et al., 1994) as children reported more cancer-related psychological stress [Impact of Events Scale (IES); Horowitz et al., 1979], more fatigue and a lower sense of well-being after tumor treatment (Fagundes et al., 2012). Breast cancer patients who completed their treatment and suffered from persistent fatigue showed a higher cumulative stress experience (STRAIN; Slavich and Epel, 2010), including childhood traumatization (CTQ; Bernstein et al., 1994), compared to patients who did not suffer from fatigue (Bower et al., 2014). The present study will combine these individual measurement instruments (GSI) which can be used as a screening instrument in clinical practice.

The DRRI-2 (Vogt et al., 2013) questionnaire is an instrument developed specifically for use with veterans. Section A is used in the GSI, which refers to life events prior to deployment. For the oncology sample, we rephrased the introduction “before my deployment” to “before my illness.” In addition, item 15 was also adapted and generally refers to difficult professional situations. However, due to time constraints, it was not possible to validate the questionnaire with the reformulated item.

Although our sample only refers to breast cancer patients and the significance for other tumor entities may be limited, all patients treated at the breast center are addressed and asked for their participation in the study. Therefore, this is a representative sample of breast cancer patients. An expansion to other tumor entities may be possible in the future.

Our longitudinal study design makes it possible to observe the progression of CRF. This gives women at the Breast Center the opportunity to undergo CRF screening over 6 months. This is the first and only opportunity at General Hospital Nuremberg to screen for CRF as the guidelines recommend (Fabi et al., 2020; Howell et al., 2015). The women may benefit from a subsequent consultation and fatigue can be improved, which future results may show.

The use of the GSI in clinical practice can define the stress risk profile of an oncological patient individually and can help identifying potential risk factors for CRF at an early stage so that treatment may be successfully addressed.

## Footnotes

^1^ https://www.danielsoper.com/statcalc/calculator.aspx?id=89
